# Preoperative limbic cortical thinning in mesial temporal lobe epilepsy surgical candidates is associated with pre‐existing and de novo postoperative depression

**DOI:** 10.1002/epd2.70297

**Published:** 2026-07-08

**Authors:** Philip Fink‐Jensen, Brice Ozenne, Ane Kloster, Bo Jespersen, Kirsten Vinter, Guido Rubboli, Martin Balslev Jørgensen, Sandor Beniczky, Melanie Ganz, Lars Hageman Pinborg

**Affiliations:** ^1^ Neurobiology Research Unit, Department of Neurology Copenhagen University Hospital Rigshospitalet Copenhagen Denmark; ^2^ Department of Public Health, Section of Biostatistics Copenhagen University of Copenhagen Copenhagen Denmark; ^3^ Department of Medical Genetics Oslo University Hospital Oslo Norway; ^4^ Department of Neurosurgery Copenhagen University Hospital Rigshospitalet Copenhagen Denmark; ^5^ Epilepsy Clinic, Member of the ERN EpiCARE Department of Neurology Copenhagen University Hospital Rigshospitalet Copenhagen Denmark; ^6^ Department of Clinical Medicine University of Copenhagen Copenhagen Denmark; ^7^ Danish Epilepsy Center, Member of the ERN EpiCARE Dianalund Denmark; ^8^ Psychiatric Center Copenhagen, Mental Health Services, Capital Region of Denmark; ^9^ Department of Neurophysiology Danish Epilepsy Center Dianalund Denmark; ^10^ Department of Neurophysiology Copenhagen University Hospital Rigshospitalet Copenhagen Denmark; ^11^ Department of Computer Science University of Copenhagen Copenhagen Denmark

**Keywords:** anterior temporal lebectomy, cortical thickness, Enigma consortium, latent variable modeling, mesial temporal lobe epilepsy, mood disorder

## Abstract

**Objective:**

Depression is common in mesial temporal lobe epilepsy (mTLE), but its neuroanatomical basis—and overlap with major depressive disorder (MDD)—remains poorly defined. We investigated whether the cortical and subcortical alterations reported in MDD are also present in mTLE and leveraged the frequent occurrence of depression after epilepsy surgery to test whether such changes predate the onset of post‐surgical depression.

**Methods:**

Eighty‐nine mTLE patients scheduled for anterior temporal lobectomy were classified as never depressed (NoD, *n* = 42), depressed before surgical evaluation (PreD, *n* = 23), or developing de‐novo depression after surgery (PostD, *n* = 22). Pre‐operative 3 T MRI (1 mm T1, T2, FLAIR) was processed with FreeSurfer v6.3. Six frontotemporal‐limbic regions showing the largest MDD effects in ENIGMA studies—orbitofrontal cortex, rostral anterior cingulate, insula, fusiform gyrus, hippocampus, posterior cingulate—were analyzed using a latent variable model.

**Results:**

Depression status significantly influenced combined morphology in both hemispheres (*p* < 0.05). Relative to NoD, both PreD and PostD groups showed reduced cortical thickness in the orbitofrontal cortex, fusiform gyrus, insula, and rostral anterior cingulate (Cohen's d = −0.04 to −0.14). Hippocampal volume and posterior cingulate thickness did not differ between groups.

**Significance:**

Four of the six frontotemporal regions showing the largest morphometric alterations in MDD also exhibit reduced cortical thickness in mTLE patients with past or future depression. This shared frontotemporal thinning pattern supports depression in mTLE as an expression of network pathology common to MDD, while residual regional differences may help explain the distinct phenotypic presentations of depression in these neuropsychiatric disorders. In PostD patients, these abnormalities were already present pre‐operatively, consistent with a pre‐existing vulnerability rather than a purely psychosocial reaction to surgery or epilepsy per se.

**Key points**

- Depression in mTLE is linked to cortical thinning in four frontotemporal regions, also altered in MDD.
- Pre‐operative frontotemporal thinning in PostD patients indicates a pre‐existing vulnerability to depression.
- Hippocampal volume and posterior cingulate thickness do not differ by depression status in mTLE.
- Depression in mTLE reflects network pathology partly shared with MDD, not merely a psychosocial reaction to epilepsy and surgery.
- Residual regional differences may help explain distinct depressive phenotypes in epilepsy versus primary mood disorder.

## 1 INTRODUCTION

Nearly 2500 years ago, Hippocrates of Kos did made the observation that “*melancholics ordinarily become epileptics, and epileptics, melancholics”*.^1^ Several registry studies have supported the concept of a bidirectional association between epilepsy and major depressive disorder (MDD).^2^, ^3^, ^4^, ^5^, ^6^ For instance, Hesdorffer et al. (2012), utilizing data from the UK General Practice Research Database, revealed a significant increase in the incidence rate ratio (IRR) of depression, anxiety, and suicidality both prior to (IRR, 1.5–15.7) and following (IRR, 2.2–10.9) an epilepsy diagnosis. Most recently, Bølling‐Ladegaard et al. (2023) reported an adjusted hazard ratio (aHR) of 1.88 for depression following epilepsy and an aHR of 2.35 for epilepsy following depression.^7^ These findings were based on a Danish nationwide register‐based matched cohort study, including all patients who received a first diagnosis of epilepsy or depression between 1980 and 2016.^7^

The fact that either condition can precede the other suggests shared pathogenic abnormalities and serves as a note of caution for clinicians who view anxiety and depression in epilepsy merely as the accumulated psychosocial consequences of the disease.^8^, ^9^ This view of depressive symptoms in epilepsy as representing shared pathogenic abnormalities is supported by findings from a recent observational and cross‐sectional study by Kanner et al. (2022), which found that 38.6% of 347 adults with newly diagnosed focal epilepsy had current or past symptoms that met the criteria for a mood and/or anxiety disorder, as assessed by the MINI International Neuropsychiatric Interview.^10^, ^11^

Morphometric features in brain MRI, such as cortical thickness and subcortical volume, are often used as structural biomarkers to study the consequences of underlying disease mechanisms in brain disorders. An example is the large cross‐sectional MRI studies conducted by the ENIGMA‐Major Depressive Disorder Working Group, which have revealed morphological alterations in cortical thickness and subcortical volume within the frontotemporal‐limbic (FTL) system in patients with major depressive disorder (MDD). The most pronounced effect sizes were observed in the orbitofrontal cortex (OFC), cingulate cortex, fusiform gyrus, insula, and hippocampus, highlighting these regions' significance in the neural pathology of MDD.^12^, ^13^ There appears to be a significant association between MDD and morphometric changes in FTL regions. However, due to the design of the studies, it is not possible to argue for a more causal relationship.

The objective of this study is to test whether we can identify and further expand on the findings observed in the ENIGMA studies in patients with MDD and epilepsy, thereby strengthening the hypothesis of common pathogenic mechanisms in patients with MDD and epilepsy. We chose to study patients with mesial temporal lobe epilepsy (mTLE) because depressive symptoms are highly prevalent in mTLE patients, affecting 30–60% of drug‐resistant mTLE patients.^14^, ^15^, ^16^, ^17^ Additionally, up to 30% of all mTLE patients develop de novo depression following epilepsy surgery, notably independent of whether the patients achieve seizure freedom postoperatively.^16^, ^18^, ^19^, ^20^, ^21^ Thus, studying this patient group allows us to examine the temporal relationship between morphometric phenotype in MRI prior to surgery and the subsequent development of depression.

We hypothesize that

1. FTL morphometrics before epilepsy surgery is lower in mTLE patients with a history of depression before surgery (PreD) compared to mTLE patients with no history of depression (NoD)
2. FTL morphometrics before epilepsy surgery is lower in mTLE patients who develop de novo depression after epilepsy surgery (PostD) compared to mTLE patients with no history of depression (NoD)

## 2 MATERIALS AND METHODS

### 2.1 Participants

We identified 124 consecutive patients with drug‐resistant mesial TLE who underwent epilepsy surgery at Rigshospitalet in Copenhagen, Denmark, between 2009 and 2021.

All patients were at least 18 years old at the time of surgery and had undergone presurgical 3 T MRI, including 3D MPRAGE T1‐weighted images with isotropic 1 mm voxel resolution and 2D TSE T2‐weighted coronal images with high in‐plane, submillimetric voxel resolution. The patients had their MRI scans evaluated for FreeSurfer analysis. We excluded patients with previous brain surgery (*n* = 5), large structural pathology (*n* = 4), and significant movement artifacts (n = 4), which could hamper the FreeSurfer analysis. The remaining 111 patients were then selected for FreeSurfer analysis, described in detail in the FreeSurfer section. The FreeSurfer output from each patient underwent quality control, and manual corrections were performed, blinded to the patients' psychiatric history. We excluded three patients due to the low quality of the FreeSurfer cortical parcellation. Psychiatric historiesy for the 108 patients were subsequently retrieved from electronic health records and databases. Sixteen patients had a lifetime psychiatric history other than depression, and three patients developed de novo anxiety disorders after surgery and were excluded from the final analysis. That left us with 89 patients for the study. This study was approved by the Danish Health Authority and the Danish Patient Safety Authority (case numbers 3–3013‐1030/1 and 3–3013‐3074/1).

### 2.2 Clinical data

Clinical patient data were retrieved from the electronic health record (EHR) of each patient. Anti‐seizure medication (ASM) use was recorded during the routine presurgical evaluation for epilepsy surgery and extracted retrospectively from the electronic health records. Medication status reflects the treatment regimen at the time of presurgical assessment and MRI acquisition.

### 2.3 Psychiatric assessment

As part of the Danish epilepsy surgery program, all patients underwent thorough pre‐ and postsurgical evaluations over a two‐year follow‐up period, conducted by a multidisciplinary team of neurologists, psychiatrists, neuropsychologists, and neurosurgeons at Rigshospitalet and the Epilepsy Hospital in Dianalund. Preoperative assessments included detailed psychiatric and neuropsychological evaluations performed by board‐certified specialists. Postoperatively, patients followed a structured program with depression screening at intervals ranging from 6 weeks to 6 months. Psychiatric symptoms were addressed either by psychiatrists affiliated with the epilepsy surgery program or by board‐certified psychiatrists in the general psychiatric care system.

Only diagnoses made by board‐certified psychiatrists, both pre‐ and post‐surgery, were considered valid. Patients diagnosed by board‐certified psychiatrists affiliated with the epilepsy surgery program met ICD‐10 criteria for a depressive disorder (F32/F33). Patients who did not fulfill the criteria for a depressive disorder but met the criteria for adjustment disorder (F43.2) were not included in the study. For patients diagnosed with depression prior to inclusion in the Danish epilepsy surgery program, clinical notes from private psychiatrists were unavailable. However, based on a thorough clinical interview, patients were categorized into the PreD group if they were deemed highly likely to have met the ICD‐10 criteria for a major depressive episode.

The 89 patients were categorized into three groups: those with a presurgical history of depression were designated PreD (*n* = 23); those without a presurgical history of depression who developed de novo depression post‐surgery were designated PostD (*n* = 24); and those with no history of depression or other psychiatric disorders either pre‐ or post‐surgery were designated NoD (*n* = 42).

### 2.4 MRI acquisition

All patient presurgical MR scans were acquired at a 3 Tesla MR scanner (Siemens, Erlangen, Germany) at Hvidovre Hospital. High‐resolution T1‐weighted images were acquired using a 3D T1‐weighted MPRAGE sequence with 1 mm isotropic resolution. This allowed for optimal contrast between gray matter, white matter, and CSF and was used as FreeSurfer input for the cortical reconstruction pipeline. In addition, T2‐weighted coronal images were added as an overlay for optimal boundary tracing accuracy. Prior to surgery, all MRIs were reviewed and described by a certified neuroradiologist.

### 2.5 FreeSurfer pre‐processing

All patients' scans were visually inspected for movement and other artifacts that could hamper the image processing. For the cortical regions and subcortical structures analysis, we used the FreeSurfer version 6.3.0 (https://surfer.nmr.mgh.harvard.edu) for the cortical parcellation and volumetric segmentation. FreeSurfer is a fully automated neuroimaging software. The downstream processing of T1‐weighted images has thirty‐one steps. In brief, this involves motion correction, transformation to Talairach space, intensity normalization, removal of non‐brain tissue, and segmentation of gray and white matter. Algorithms and a smoothing process are then applied to correct for topological defects. Each hemisphere's cortical surface is then inflated and registered into a spherical atlas, allowing each hemisphere's cortical surface to be parcellated into thirty‐four distinct neuroanatomical regions of interest (ROI) based on an atlas proposed by Desikan et al.^22^ The cortical reconstructions were then checked and corrected manually for surface displacements. The same person performed all manual corrections to ensure consistency in the data set, blinded to patients' identity and depression status. The cortical thickness is measured as the shortest distance between the gray/white matter junction and the pial surface at each vertex.^23^ Two PostD patients had unsuccessful segmentation results of the insular cortex and were excluded from all remaining analyses, thus resulting in a total of 22 PostD patients.

### 2.6 Statistical modeling

To investigate the association between cortical and subcortical morphological alterations in mTLE patients, we applied a linear latent variable model using the Lava package in R.^24^ A linear latent variable model is a multivariate regression framework that allows researchers to relate a group variable (i.e., depression) to multiple morphological characteristics (e.g., cortical thickness and subcortical volume) via a latent variable. Instead of modeling the exposure effect on each morphological characteristic individually, the effect is mediated through the latent variable (see Figure 1). The latter can be understood as a summary of the morphological characteristics, and was hence coined FTL morphology. We investigated whether the average morphological measurements (e.g., cortical thickness and subcortical volume) differed between PreD patients and NoD patients or between PostD patients and NoD patients. A separate model was constructed for each hemisphere. “Ipsilateral” referred to the hemisphere where the epileptogenic zone was identified before operation, while “contralateral” referred to the opposite hemisphere. Hippocampal volumes were measured bilaterally and analyzed relative to the epileptogenic hemisphere (ipsilateral vs. contralateral). Each model contained a single latent variable defined based on the average thickness of the orbitofrontal cortex (OFC), fusiform gyrus (FG), insula (INS), rostral anterior cingulate cortex (raCC), and posterior cingulate cortex (pCC), as well as the volume of the hippocampus (HIP). These regions were selected based on those identified by the ENIGMA‐Major Depressive Disorder Working Group^12^, ^13^ specifically the regions that bilaterally showed a significant effect after false discovery rate correction for the difference between healthy individuals and MDD patients.

**FIGURE 1:**
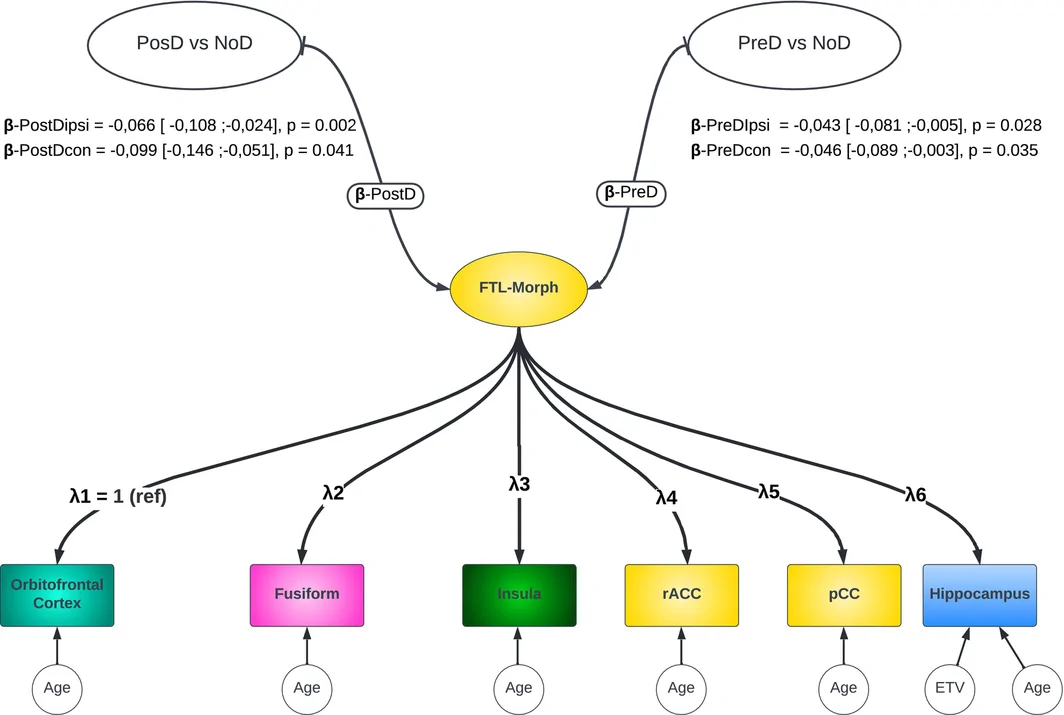
Latent Variable Modeling of FTL Morphology. The latent variable model shows the relationship between the latent factor “FTL‐Morph” and morphological measures of six brain regions. Group differences (PreD vs. NoD, PostD vs. NoD) are depicted as path coefficients (β) with confidence intervals and *p*‐values for ipsilateral and contralateral hemispheres. Structural coefficients (λ2 to λ6) represent the contribution of “FTL‐Morph” to each region, with age as a covariate and ETV specific to hippocampal volume. Significant group effects were observed for both hemispheres.

In the latent variable model, cortical regions were adjusted for age (see figure) whilst subcortical regions (e.g., hippocampus) were adjusted for both age and intracranial volume to account for potential. Intracranial volume was quantified using SPM12 (v7219, https://www.fil.ion.ucl.ac.uk/spm) in Matlab (R2019a, MathWorks, Natick, MA, USA) by segmenting the gray matter, white matter, and cerebrospinal fluid and summing their combined volume.^25^ As a sensitivity analysis, we re‐fitted the latent variable model by including age of onset as an additional covariate for both cortical and subcortical regions. The estimated group differences from these models were compared with those from the original analysis, which adjusted for age but not age of onset. Epilepsy duration was not included, as it is directly dependent on age at surgery and age of onset (epilepsy duration = age at surgery − age at onset).

## 3 RESULTS

### 3.1 Demographic and clinical characteristics

The demographic and clinical characteristics of the patient groups are summarized in Table 1. In the PostD group, the median time from surgery to depression diagnosis was 18 weeks (range: 3–178 weeks). Among these patients, 19 received antidepressant treatment, while 5 underwent psychotherapy alone or declined treatment. Clinical and demographic data were analyzed across all three groups. No statistically significant differences in demographic and clinical data were observed between the groups, as detailed in Table 1. In particular, there were no differences in seizure outcomes or the use of anti‐seizure medications between groups. The most common MRI abnormality reported by the two neuroradiologists associated with the Danish epilepsy surgery program was mesial temporal lobe sclerosis, followed by malformations of cortical development and neuroepithelial tumors. Seventeen per cent of scans showed no MRI abnormalities. There was no statistically significant difference in MRI abnormalities between the groups (Chi‐square test, *p* > 0.24).

**TABLE 1:** Demographic and clinical characteristics of epilepsy surgery patients.

Demographic and surgical data	NoD (*n* = 42)		PostD (*n* = 22)		PreD (*n* = 23)		*p*‐value
	Median, *n*	Range, %	Median, *n*	Range, %	Median, *n*	Range, %	
Age at surgery (years)	33	24–58	42	18–67	43	21–63	0.08^b^
Age at first seizure (years)	16	1–51	18	1–53	17	0–56	0.32^b^
Time from first seizure to surgery (years)	14	5–45	22	0–54	14	0–51	0.61^b^
Gender (female)	20	47.6%	14	63.6%	12	52.2%	0.47^a^
Seizure‐free 2 years post operation	26	61.9%	16	72.7%	19	82.6%	0.21^a^
Surgery hemisphere (left)	26	61.9%	11	50.0%	13	56.5%	0.65^a^

ASM before surgery	Number of patients	Range, %	Number of patients	Range, %	Number of patients	Range, %	*p*‐value
Sodium valproate	4	9.5	3	13.6	0	0	0.22^a^
Lamotrigine	18	42.9	12	54.5	9	39.1	0.55^a^
Carbamazepine	5	11.9	3	13.6	2	8.7	0.87^a^
Zonisamide	7	16.7	8	36.4	2	8.7	0.05^a^
Lacosamide	21	50.0	14	63.6	7	30.4	0.08^a^
Topiramate	10	23.8	4	18.2	3	13.0	0.57^a^
Levetiracetam	16	38.1	8	36.4	12	52.2	0.46^a^
Perampanel	3	7.1	1	4.5	1	4.3	0.86^a^
Clobazam	4	9.5	7	31.8	5	21.7	0.08^a^
Oxcarbazepine	7	16.7	1	4.5	1	4.3	0.17^a^
Eslicarbazepine acetate	2	4.8	0	0	4	17.4	0.05^a^

Presurgical MRI diagnosis	Number of patient	Range, %	Number of patients	Range, %	Number of patients	Range, %	*p*‐value
Mesial temporal lobe sclerosis	17	40,5	12	54,5	6	26,1	0.36^a^
Malformation of cortical development	4	9,5	2	9	2	8,7	0.99^a^
Tumor	4	9,5	1	4,5%	2	8,7	0.78^a^
Other Pathology	1	2,4	0	0	2	8,7	0.24^a^
MRI negative	7	16,7	3	13,6	5	21, 7	0.76

^a^ Chi‐square test.

^b^ Kruskal Wallis test.

### 3.2 Overall effects of depression status on FTL morphology

In the ipsilateral hemisphere, depression status was associated with a significant reduction in average FTL morphology for both the PreD group (Estimate = −0.043, 95% CI [−0.081; −0.005], *p* = 0.028) and the PostD group (Estimate = −0.066, 95% CI [−0.108; −0.024], *p* = 0.002), as illustrated in Figure 2, which presents the distribution of cortical thickness and hippocampal volume across groups. In the contralateral hemisphere, a similar pattern was observed, where both groups showed a significant reduction in average FTL morphology, although the reduction was greater in the PostD group (Estimate = −0.099, 95% CI [−0.146; −0.051], *p* < 0.001) than in the PreD group (Estimate = −0.046, 95% CI [−0.089; −0.003], *p* = 0.035).

**FIGURE 2:**
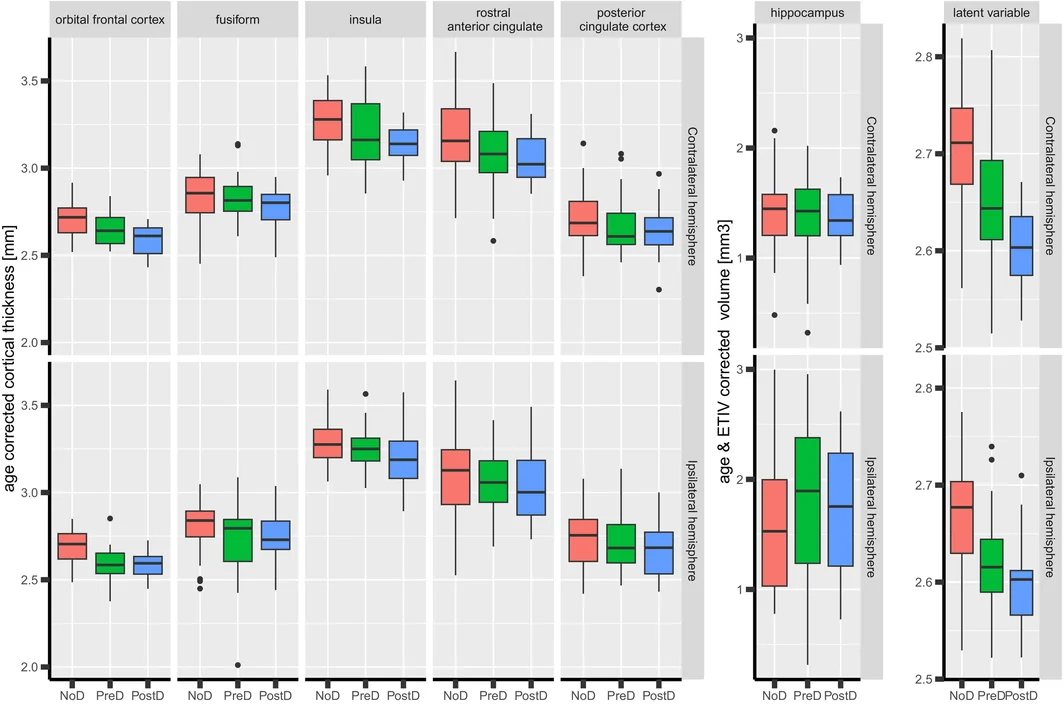
Boxplots of cortical thickness and latent FTL‐morphology. The boxplots to the left show the age‐corrected cortical thickness for the selected ROIs. The boxplot to the right shows the distribution group differences in the latent variable, i.e., FTL‐morphology. The latent variable summarizes the region‐specific differences across all regions (i.e., regions to the left of the latent variable).

Tables 2 and 3, along with Figure 2, illustrate how group differences in FTL morphology manifest at the individual region level. At a significance threshold of *p* = 0.05, cortical thickness in the orbitofrontal cortex, fusiform gyrus, insula, and rostral anterior cingulate cortex was significantly reduced in both the PreD and PostD groups compared to the NoD group. No significant group differences were observed for cortical thickness in the posterior cingulate cortex or for hippocampal volume. After applying a Bonferroni correction for multiple comparisons (across six regions, ipsilateral and contralateral to the operated hemisphere), yielding a corrected significance threshold of *p* < 0.0042, only the aforementioned four cortical regions remained significantly different between the PostD and NoD groups.

**TABLE 2:** Effects of depression status on cortical thickness for patients with a previous history of depression (PreD) compared to patients without a history of depression (NoD)—adjusted for age.

Anatomical region	Ipsilateral to surgery				Contralateral to surgery			
	Estimate	Unit	95% CI	*p*‐value	Estimate	Unit	95% CI	*p*‐value
Orbitofrontal Cortex	−0,043	mm	[−0,081;−0,005]	0,028	−0,046	mm	[−0,089;−0,003]	0,035
Fusiform Cortex	−0,065	mm	[−0,124;−0,006]	0,031	−0,049	mm	[−0,095;−0,002]	0,039
Insula Cortex	−0,064	mm	[−0,120;−0,008]	0,025	−0,062	mm	[−0,120;−0,003]	0,038
Ros. Anterior Cingulate Cortex	−0,083	mm	[−0,158;−0,008]	0,031	−0,064	mm	[−0,126;−0,002]	0,045
Posterior Cingulate Cortex	−0,055	mm	[−0,105;−0,004]	0,034	−0,016	mm	[−0,042;0,010]	0,223
Hippocampus	−0,058	mm^3^	[−0,166;0,050]	0,292	−0,042	mm^3^	[−0,099;0,017]	0,161

**TABLE 3:** Effects of depression status on cortical thickness for patients with de novo depression after surgery (PostD) compared to patients without a history of depression (NoD)—adjusted for age.

Anatomical region	Ipsilateral to surgery				Contralateral to surgery			
	Estimate	Unit	95% CI	*p*‐value	Estimate	Unit	95% CI	*p*‐value
Orbitofrontal Cortex	−0,066	mm	[−0,108;−0,024]	0,002	−0,099	mm	[−0,146;−0,051]	0,0000
Fusiform Cortex	−0,099	mm	[−0,165;−0,034]	0,003	−0,103	mm	[−0,157;−0,050]	0,0002
Insula Cortex	−0,098	mm	[−0,157;−0,038]	0,001	−0,131	mm	[−0,198;−0,065]	0,0001
Ros. Anterior Cingulate Cortex	−0,127	mm	[−0,211;−0,043]	0,003	−0,136	mm	[−0,212;−0,059]	0,0005
Posterior Cingulate Cortex	−0,084	mm	[−0,141;‐0,027]	0,004	−0,034	mm	[−0,082;0,014]	0,161
Hippocampus	−0,089	mm^3^	[−0,244;0,067]	0,263	−0,088	mm^3^	[−0,189;0,013]	0,088

#### 3.2.1 Post hoc analysis of the association between age, age of onset, and cortical thickness

A sensitivity analysis was conducted to re‐estimate the group effect on cortical thickness after additional adjustment for age at epilepsy onset and age at surgery, compared with the core model adjusting for age only. Differences in point estimates between the two models were small, ranging from −2% to 2% in the PostD vs. NoD comparison and from −2% to 4% in the PreD vs. NoD comparison. In the PostD vs. NoD comparison, the largest difference was observed in the ipsilateral OFC (0.0013 mm; 2%). The direction and statistical significance of the effects were consistent across all comparisons (see Tables S1 and S2).

## 4 DISCUSSION

We set out to investigate the relationship between FTL morphometry and depression in mTLE patients. Based on large cross‐sectional MRI studies in MDD patients conducted by the ENIGMA‐Major Depressive Disorder Working Group, we selected five cortical regions (orbitofrontal cortex, rostral anterior cingulate, posterior cingulate, insula, and fusiform gyrus) and one subcortical region (hippocampus) that demonstrated the largest Cohen's d effect sizes in the ENIGMA studies.^12^, ^13^ Our study demonstrates an overlap in cortical thinning within FTL networks between mTLE patients with depression and the alterations reported in MDD by the ENIGMA consortium. This suggests a shared anatomical substrate while also highlighting differences in the hippocampus and posterior cingulate that may underlie the distinct phenotypic presentations of these neuropsychiatric disorders. Notably, in light of the high incidence of post‐operative depression after temporal‐lobe surgery, our data indicate that these morphologic changes likely precede the emergence of depressive symptoms.

### 4.1 Analyses of data using the linear latent variable model

We tested whether mTLE patients with prior depression show frontotemporal–limbic (FTL) morphometric alterations relative to never‐depressed mTLE patients. Using a linear latent‐variable model,^24^ we assessed the combined effect of depression on the six regions with the largest effect sizes in ENIGMA‐MDD studies.^12^, ^13^ This multivariate approach boosts power with our modest sample. The analysis confirmed that the same six regions are likewise abnormal in mTLE patients with a history of depression.

In both PreD and PostD groups, all regions of interest were significantly altered, except for the hippocampus and the posterior cingulate cortex contralateral to the surgical side; after Bonferroni correction, the same two regions remained non‐significant in PostD. Accordingly, our dataset shows no association between hippocampal volume and depression in mTLE. This apparently counter‐intuitive result aligns with reports that hippocampal sclerosis is not linked to depression in epilepsy.^26^ The ENIGMA‐MDD consortium did find smaller hippocampi in MDD, but that effect was driven mainly by patients with recurrent episodes^13^—conditions absent in our PostD sample and unlikely in PreD candidates referred for surgery. Although we lack detailed longitudinal psychiatric histories, severe recurrent depression would probably have excluded patients from surgical work‐up. Finally, wide confidence intervals, reflecting varied degrees of hippocampal sclerosis, prevent us from dismissing modest yet clinically relevant hippocampal differences. Whilst the algorithm used to delineate the hippocampal segmentation was based on non‐epileptic patients, automated hippocampal segmentation has previously been shown to correspond with histopathological findings in hippocampal sclerosis, supporting the validity of hippocampal volume measurements in patients with HS.^27^

### 4.2 How does our study add to a better understanding of de novo depression after epilepsy surgery

PostD patients developed depression a median of 4 months after surgery, mirroring previous studies,^21^, ^28^, ^29^ underscoring the mesial and anterior temporal lobe's importance in depression onset. All but one patient underwent an anterior temporal lobectomy (Spencer's technique^30^), which removes or lesions the anterior hippocampus, amygdala, and their prefrontal connections via the uncinate fasciculus. Yet this intervention alone did not trigger depression: only those who later developed de‐novo depression showed pre‐surgical thinning in orbitofrontal, anterior cingulate, and insular cortices—regions remote from the epileptogenic zone—whereas seizure‐free NoD patients did not. We therefore propose that pre‐existing structural alterations in the wider frontotemporal‐limbic network, rather than resection of mesial temporal tissue per se, shape network topology and create a vulnerability that facilitates the emergence of depressive symptoms post‐operatively.

### 4.3 How does our study add to a better understanding of epilepsy in general?

Our study supports the understanding of epilepsy as a condition characterized by widespread morphological and functional changes in the brain, extending beyond a localized disorder confined to the epileptogenic zone and its associated seizure activity. Specifically, we show how the group of mTLE patients with a history of depression, compared to patients with no history of depression, exhibit lower regional cortical thickness in the FTL networks across both hemispheres known to be important for mood regulation. This insight underscores the critical importance of systematically screening epilepsy patients for psychiatric disorders, not only in the context of epilepsy surgery but also, and perhaps more importantly, at the time of their first seizure.^31^, ^32^ Early identification of psychiatric comorbidities, such as depression and anxiety, is essential to providing comprehensive care and addressing the broader impact of epilepsy on mental health.^33^

### 4.4 How does our study contribute to a better common understanding of epilepsy and MDD?

Knowledge about the temporal relationship between morphological changes and clinical symptoms is unavailable from studies of brain morphology in MDD, which rely on a cross‐sectional design. Our study reinforces the notion that disruptions within the FTL network play a causal role in the emergence of depressive symptoms, underscoring the network's pivotal involvement in mood regulation.

Focal onset epilepsy can arise from a variety of aetiologies but shares the common feature that at some point during epileptogenesis, abnormal electrical activity is generated spontaneously from the epileptogenic zone. We hypothesize that this hypersynchronized electrical activity propagates through local structural networks, progressively inducing reorganization of global functional networks, particularly in mTLE patients who are involved in cognition and mood regulation. This reorganization likely explains why many epilepsy patients exhibit cognitive and psychiatric symptoms at the time of their first epileptic seizure or even before.^10^, ^34^ In that sense, mTLE may serve as an illustrative example of what Northoff & Hirjak (2024), in their perspective article on MDD, refer to as “topographic dynamic reorganization”.^35^ Changes in excitation‐inhibition balance may indeed provide a clue to the shared pathogenic pathways underlying the bidirectional relationship between epilepsy and depression.

While epilepsy patients with mood disorders share many similarities with mood disorders in individuals without epilepsy, they also present intriguing differences. For instance, many patients experience years of chronic depressive symptoms or dysthymia, dominated by low mood and irritability as prominent features, significantly compromising their daily quality of life.^36^, ^37^ However, these symptoms often fluctuate, with days of distress alternating with days of minimal or no symptoms,^38^ and as a result, these patients may not formally meet the DSM‐5 or ICD‐11 criteria for either MDD or Persistent Depressive Disorder. However, patients endure significant emotional distress and functional impairment and benefit from appropriate treatment.^9^ It is also well‐established that some patients with epilepsy experience dysphoria in the days leading up to a seizure, and many patients frequently report episodes of depressive symptoms or a worsening of pre‐existing depressive symptoms in the period following a seizure.^38^ We speculate that the persistent load of hypersynchronized abnormal interictal and episodic ictal activity from the epileptogenic zone into FTL networks leads to a “topographic dynamic reorganization” of the epileptic brain, which may explain both the fluctuating and persistent aspects of the symptomatology of mood disorders in epilepsy.

### 4.5 Consistency of findings with prior studies

Depression in epilepsy often presents atypically^37^ and may be hard to capture retrospectively. By integrating comprehensive psychiatric monitoring and assessments by board‐certified psychiatrists within the Danish Epilepsy Surgery Program, we aim to minimize the risk of patient misclassification. In our cohort, 26% had pre‐surgical depression, closely matching the pooled 25% prevalence (range 0–40%) from 16 TLE surgery studies.^38^ De novo post‐surgical depression occurred in 25% of our patients, in line with reports of 0–38% across 11 studies with 3‐ to 9‐year follow‐up^38^.

### 4.6 Strengths, limitations, and future directions

To the best of our knowledge, this cohort represents by far the largest study to date investigating depressive symptoms in relation to brain morphology in mTLE patients, pre‐ and post‐epilepsy surgery.

However, we fully acknowledge the *limitation* posed by the sample sizes of the three subgroups, which inherently reduce statistical power and increase the risk of Type II errors. However, we emphasize that our findings align with an a priori hypothesis, grounded in previous ENIGMA large‐scale studies in MDD patients. Additionally, the PostD results remain significant even after rigorous statistical testing using Bonferroni correction.

A key *strength* of our study lies in the homogeneity of our cohort, consisting of 87 patients, all diagnosed with drug‐resistant mTLE upon inclusion in the Danish epilepsy surgery program. None of the patients had been previously diagnosed with, nor were subsequently diagnosed with, any psychiatric disorders other than depression. There was no significant difference between the PreD, PostD, and NoD groups in demographic variables, pre‐surgical or post‐surgical variables (including surgical outcome). Throughout both the pre‐surgical evaluation process and the post‐surgical follow‐up program, all patients were assessed by the same multidisciplinary teams, including board‐certified psychiatrists conducting psychiatric interviews.

With the exception of one patient, all individuals underwent an anterior temporal lobectomy performed by the same neurosurgical team. Furthermore, all MRI scans and FreeSurfer results were rigorously quality‐assessed by our team, recognized as a European training site for FreeSurfer analysis of structural MRIs. This team maintains a longstanding collaboration with the developers of FreeSurfer at the Martinos Center for Biomedical Imaging at Massachusetts General Hospital in Boston.

A *limitation* of our study is that the PreD, PostD, and NoD groups were identified retrospectively based upon the structured clinical interviews conducted by board‐certified psychiatrists at inclusion and during the course of the epilepsy surgery program. Patients in the PreD and PostD groups fulfilled ICD‐10 criteria for depressive disorder (F32/F33). Stratifying patients according to depression severity could provide additional insight into potential associations between cortical thickness and depressive symptom burden. However, data on symptom severity and duration were not available.

In addition, we did not have access to information on ASM use in relation to the timing of depression onset in the PreD group. Thus, antiseizure medications, particularly levetiracetam, may represent a potential confound given the close relationship between ASM exposure and mood regulation. However, there was no association between the timing of depression and ASM use in the PostD group, and the similar distribution of ASM exposure across groups makes it less likely that ASM treatment alone accounts for the observed morphometric differences. The neurobiological mechanisms underlying interindividual susceptibility to affective symptoms following ASM exposure remain poorly understood.

Another potential confounder relates to the use of antidepressant medication. Neuroimaging studies have suggested that SSRI treatment may induce structural brain changes,^39^, ^40^ including increases in cortical thickness in frontotemporal regions, particularly within the orbitofrontal cortex, potentially reflecting treatment‐related neuroplasticity. In the present cohort, antidepressant exposure was confined to the PreD group, with six patients receiving SSRI treatment at the time of MRI acquisition. This exposure would be expected to attenuate group differences and thus bias the results towards the null, suggesting that the observed differences may represent conservative estimates of the true effect.

Etiological heterogeneity within mTLE (e.g., mesial temporal sclerosis, focal cortical dysplasia, tumors, and MRI‐negative epilepsy) may influence cortical morphometric patterns. Although we did not observe significant differences in the distribution of MRI‐defined pathologies across the three groups, we cannot fully exclude an effect of underlying pathology on cortical measures. Larger multicentre studies will be required to disentangle the contribution of specific aetiologies.

Finally, a limitation of this study is the absence of external control groups, including both healthy controls and patients with MDD, which precludes direct comparison with established morphometric patterns in depression. However, the primary aim was to identify neuroanatomical correlates of depression within epilepsy, and we therefore employed an internally controlled design comparing patients with epilepsy with and without depression. Given the modest sample size and the rarity of the surgical epilepsy population, we further applied a hypothesis‐driven region‐of‐interest approach based on regions identified in large‐scale ENIGMA MDD studies, thereby reducing the multiple comparisons burden and increasing statistical power. Future large‐scale international studies combining epilepsy and psychiatric cohorts will be important to determine the extent to which the observed patterns overlap with those seen in MDD and whether potential differences relate to distinct clinical manifestations of depressive symptoms in patients with epilepsy compared with patients with MDD.

## FUNDING INFORMATION

PFJ was supported by the Danish Neurological Society and the Lundbeck Foundation. AK was supported by the Research Counsil of Rigshospitalet. LHP was supported by the Lundbeck Foundation, the Svend Andersen Foundation, and the Stockbroker Henry Hansen's and Wife Karla Hansen's Foundation. The study was supported by the Lundbeck Foundation (BrainDrugs, Grant ID: R279‐2018‐1145).

## CONFLICT OF INTEREST STATEMENT

The authors declare no conflicts of interest.

**Test Yourself**

1. Which pattern best describes the main morphometric finding in relation to depression status?
  - Larger hippocampal volumes and thicker posterior cingulate cortex in PreD and PostD vs. NoD.
  - Reduced cortical thickness in orbitofrontal cortex, fusiform gyrus, insula, and rostral anterior cingulate in PreD and PostD vs NoD, with no group differences in hippocampal volume or posterior cingulate.
  - Global cortical atrophy and reduced intracranial volume in all depressed patients vs. NoD.
  - Structural abnormalities confined to the ipsilateral hippocampus in PreD and PostD vs. NoD.
2. What is the main implication of the finding that PostD patients already showed FTL cortical thinning pre‐operatively?
  - Post‐surgical depression is most likely a direct pharmacological side effect of anti‐seizure medications.
  - Depression after surgery is best understood as a transient psychological reaction that resolves spontaneously.
  - There is a pre‐existing network vulnerability within frontotemporal–limbic circuits that likely precedes and facilitates the emergence of depressive symptoms after surgery.
  - Mesial temporal resection alone is sufficient to induce de novo depression in all mTLE patients.
3. How does this study advance the broader understanding of the relationship between epilepsy and MDD?
  - By showing that depression in epilepsy is unrelated to brain structure and purely psychosocial.
  - By demonstrating that ENIGMA‐MDD frontotemporal–limbic alterations overlap with depression‐related changes in mTLE, while differences in the hippocampus and posterior cingulate may help explain distinct depressive phenotypes.
  - By proving that hippocampal atrophy is the sole common substrate of epilepsy and MDD.
  - By establishing that only patients with recurrent MDD episodes should be considered for epilepsy surgery.

*Answers may be found in the* *supporting information*.

## Supporting information


**Table S1.** Effects of depression status on cortical thickness for patients with a previous history of depression (PreD) compared to patients without a history of depression (NoD), adjusted for age and age of onset.

**Table S2.** Effects of depression status on cortical thickness for patients with de novo depression after surgery (PostD), compared to patients without a history of depression (NoD), adjusted for age and age of onset.

Data S1.

## Data Availability

The data that support the findings of this study are available on request and an appropriate data‐sharing agreement. The data are not publicly available due to privacy or ethical regulatory restrictions.
