# Supplementary material for: Preoperative limbic cortical thinning in mesial temporal lobe epilepsy surgical candidates is associated with pre‐existing and de novo postoperative depression
Source: Epileptic Disord. 2026 Jul 8;28(5):1554–66. doi: 10.1002/epd2.70297 (PMC13695479; doi:10.1002/epd2.70297)
Supplement: Supplementary file 1 [file EPD2-28-1554-s001.docx]

**Table 4. The effects of depression status on cortical thickness for patients with a previous history of depression (PreD) compared to patients without a history of depression (NoD), adjusted for age and age of onset**

| **Anatomical region** | **Ipsilateral to surgery** | | | | **Contralateral to surgery** | | | |
| --- | --- | --- | --- | --- | --- | --- | --- | --- |
| **Anatomical region** | **Estimate** | **Unit** | **95% CI** | **p-value** | **Estimate** | **Unit** | **95% CI** | **p-value** |
| Orbitofrontal Cortex | -0.0447 | mm | [-0.0834 ; -0.0059] | 0.0237941 | -0.0462 | mm | [-0.0894 ; -0.0030] | 0.0361005 |
| Fusiform Cortex | -0.0668 | mm | [-0.1261 ; -0.0075] | 0.0272461 | -0.0493 | mm | [-0.0963 ; -0.0022] | 0.0400326 |
| Insula Cortex | -0.0657 | mm | [-0.1217 ; -0.0098] | 0.0213325 | -0.0613 | mm | [-0.1196 ; -0.0030] | 0.0394391 |
| Ros. Anterior Cingulate Cortex | -0.0848 | mm | [-0.1602 ; -0.0093] | 0.0277172 | -0.0636 | mm | [-0.1262 ; -0.0011] | 0.0459899 |
| Posterior Cingulate Cortex | -0.0560 | mm | [-0.1067 ; -0.0052] | 0.0306292 | -0.0157 | mm | [-0.0410 ; 0.0097] | 0.2260160 |
| Hippocampus | -0.0634 | mm3 | [-0.1733 ; 0.0465] | 0.2579535 | -0.0401 | mm3 | [-0.0972 ; 0.0170] | 0.1687808 |
