# Supplementary material for: Preoperative limbic cortical thinning in mesial temporal lobe epilepsy surgical candidates is associated with pre‐existing and de novo postoperative depression
Source: Epileptic Disord. 2026 Jul 8;28(5):1554–66. doi: 10.1002/epd2.70297 (PMC13695479; doi:10.1002/epd2.70297)
Supplement: Supplementary file 2 [file EPD2-28-1554-s002.docx]

**Table 5. The effects of depression status on cortical thickness for patients with de novo depression after surgery (PostD) compared to patients without a history of depression (NoD) adjusted for age and age of onset**

| **Anatomical region** | **Ipsilateral to surgery** | | | | **Contralateral to surgery** | | | |
| --- | --- | --- | --- | --- | --- | --- | --- | --- |
| **Anatomical region** | **Estimate** | **Unit** | **95% CI** | **p-value** | **Estimate** | **Unit** | **95% CI** | **p-value** |
| Orbitofrontal Cortex | -0.0673 | mm | [-0.1092 ; -0.0254] | 0.0016349 | -0.0983 | mm | [-0.1454 ; -0.0513] | 4.235e-05 |
| Fusiform Cortex | -0.1007 | mm | [-0.1662 ; -0.0352] | 0.0025942 | -0.1048 | mm | [-0.1589 ; -0.0508] | 0.0001431 |
| Insula Cortex | -0.0991 | mm | [-0.1585 ; -0.0396] | 0.0010939 | -0.1304 | mm | [-0.1970 ; -0.0639] | 0.0001218 |
| Ros. Anterior Cingulate Cortex | -0.1278 | mm | [-0.2114 ; -0.0442] | 0.0027429 | -0.1355 | mm | [-0.2120 ; -0.0589] | 0.0005232 |
| Posterior Cingulate Cortex | -0.0844 | mm | [-0.1414 ; -0.0273] | 0.0037543 | -0.0333 | mm | [-0.0802 ; 0.0135] | 0.1631627 |
| Hippocampus | -0.0956 | mm3 | [-0.2508 ; 0.0596] | 0.2274801 | -0.0853 | mm3 | [-0.1857 ; 0.0150] | 0.0956164 |
