# Supplementary material for: Preoperative limbic cortical thinning in mesial temporal lobe epilepsy surgical candidates is associated with pre‐existing and de novo postoperative depression
Source: Epileptic Disord. 2026 Jul 8;28(5):1554–66. doi: 10.1002/epd2.70297 (PMC13695479; doi:10.1002/epd2.70297)
Supplement: Supplementary file 3 [file EPD2-28-1554-s003.docx]

**Test yourself**

**Answers:**

1-B; 2-C; 3-B
